# Dental developmental alterations in patients with dilacerated teeth

**DOI:** 10.4317/medoral.22698

**Published:** 2018-12-24

**Authors:** Constantino Ledesma-Montes, María-Dolores Jiménez-Farfán, Juan-Carlos Hernández-Guerrero

**Affiliations:** 1 Clinical Oral Pathology Laboratory. Facultad de Odontología. División de Estudios de Posgrado. Universidad Nacional. Autónoma de México. Ciudad de México. 04510. MÉXICO; 2 Laboratory of Immunology. Facultad de Odontología. División de Estudios de Posgrado. Universidad Nacional. Autónoma de México. Ciudad de México. 04510. MÉXICO

## Abstract

**Background:**

The aim of this study was to record and analyze all DDAs associated to dilacerated teeth in patients attending the clinics of the Postgraduate Division, Facultad de Odontología, UNAM in Mexico City.

**Material and Methods:**

Orthopantomograms from all patients seeking for stomatological attention in our institution were reviewed and those cases of dilaceration were separated. Age, gender, diagnosis, location, involved teeth and associated DDAs were recorded and analyzed.

**Results:**

From 6,340 patients, 99 (1.6%) harbored 125 dilacerated teeth. Of them, 45 (45.5%) showed one or more DDAs. The most frequently detected DDAs were hypodontia, enamel pearls, taurodontism and microdontia.

**Conclusions:**

45.5% is a very high proportion of DDAs in patients with dilacerated roots. Findings from this study strongly suggest that patients with dilacerated teeth should be carefully screened since many of them could present other DDAs. Simultaneous occurrence of dilaceration and DDAs suggests synchronic developmental defects during dental growth.

** Key words:**Developmental alterations; dental developmental alterations; root dilaceration.

## Introduction

In 1849, Tomes described a rarely observed developmental alteration of the dental root now known as dilaceration (1). The most recent edition of the Glossary of Endodontic Terms of the American Academy of Endodontists, the dilaceration of the dental roots was defined as “a deformity characterized by displacement of the root from its normal alignment with the crown; may be a consequence of injury during tooth development. Common usage has extended the term to include sharply angular or deformed roots” (2). Two possible etiological causes of dilaceration were suggested: trauma to the primary tooth resulting in displacement of the tooth germ (2) and to a developmental disturbance of unknown origin when a traumatic factor is not known (3).

In a previous study, we reported the frequency of dilacerated teeth in the population attended in our institution (4). During the review of these radiographic material we were able to detect that several cases of dilacerated teeth were associated with other dental developmental alterations (DDAs). Taking in count this observed association, we considered that it was mandatory to make a more stringent review of these radiographs in order to detect more cases. Recognition of multiple associated pathologies on all the radiographic material from each attended patient is mandatory, and it is important to make a precise diagnosis for planning the better treatment.

Our review of the literature revealed that no data on the association of DDAs in patients with dilacerated teeth was published to date. For this reason, the aim of this study was to record and analyze all DDAs associated to dental root dilacerations in the patients attending to our institution.

## Material and Methods

This study included all the patients who sought stomatological attention during one year in the Admission and Diagnosis Clinic of the División de Estudios de Posgrado e Investigación, Facultad de Odontología-UNAM in Mexico City and a Letter of Consent was signed by patients or parents. Also, the protocol was reviewed and approved by the Ethics Committee of our institution. A panoramic radiograph was made to all patients and their radiographs were reviewed and discussed by the panel. Also, the patients were asked if any parent or relative were affected with a syndrome or any other related disease.

A dilacerated tooth was diagnosed when the mesial or distal direction of the root formed a 90° or greater angle along the axis of the tooth or root. Orofacial direction of the dilacerations was determined by evaluating the bull’s eye appearance of the root, which results from the root deviation of 90º or more. In multirooted teeth, a tooth was recognized as having dilaceration if at least one root was dilacerated, and it was counted as one case. To diagnose dilaceration, the affected tooth must be fully developed. Additionally, all DDAs in patients with dilacerated teeth were recorded and their ipsilateral or contralateral position was recorded too.

Before starting the investigation, intra-examiner calibration was done by reading 100 radiographs including cases of dilacerated teeth. Two weeks and one month after the first examination, the examiners read an extra-sample of 100 panoramic radiographs containing dilacerations and a 100% agreement was obtained. Documented data were age, gender, diagnosis, location and involved teeth. They were analyzed by SPSS (v13), the Student T test was applied and *p*< 0.05 was considered statistically significant. Data on developmental alterations occurring in third molars were not included.

## Results

From the 6,340 patients attending our clinic, we detected 99 patients (1.6%) harboring 125 dilacerated teeth. 123 teeth were of the permanent formula (98.4%) and 2 were deciduous teeth (1.6%). Of the 99 patients, there were 68 females (68.7%) and 31 males (31.3%) and statistical significance was found (*p*<0.001). Patients ages were between 7 and 80 years (mean age= 39 years). Of them, 45 patients (45.5%) showed radiographic evidence of one or more DDAs. They were 17 men (37%) and 28 women (63%). This difference was statistically significant (*p*<0.05). In these patients we detected 87 DDAs. The most frequent DDAs were hypodontia (25.3%), enamel pearls (16.1%) and taurodontism (15%) comparing these data, statistical significance was found (*p*<0.05). Data on frequency of DDAs is in  Table 1 and data on location is in  Table 2. Sixty-five DDAs were in maxilla (74.7%), and 22 in mandible (25.3%). Statistical significance was found (*p*<0.05). From the diagnosed DDAs, 40 were in the anterior portion (44.8%), 36 were detected in the molar zone (41.4%) and 11 were in the premolar area (12.6%). Comparison between location of the analyzed entities was statistically significant (*p*< 0.05). Ipsilateral position of the DDA with respect to the dilacerated tooth was found in 49 cases (56.3%) and contralateral location was detected in 38 patients (43.7%) and statistical significance was not found (*p*>0.05). 85 DDAs were associated to permanent dentition (97.7%) and two were in relation to deciduous teeth (2.3%). Difference between both data was statistically significant too (*p*<0.001). No data on traumatic events in the area was recorded.

**Table 1:**
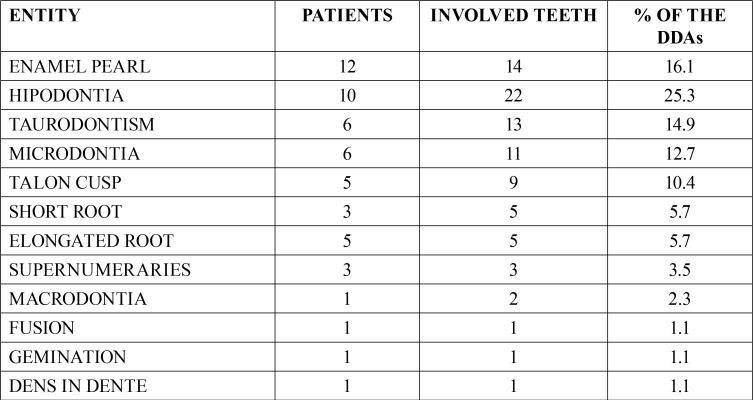
Dental developmental alterations associated to dilaceration.

**Table 2:**
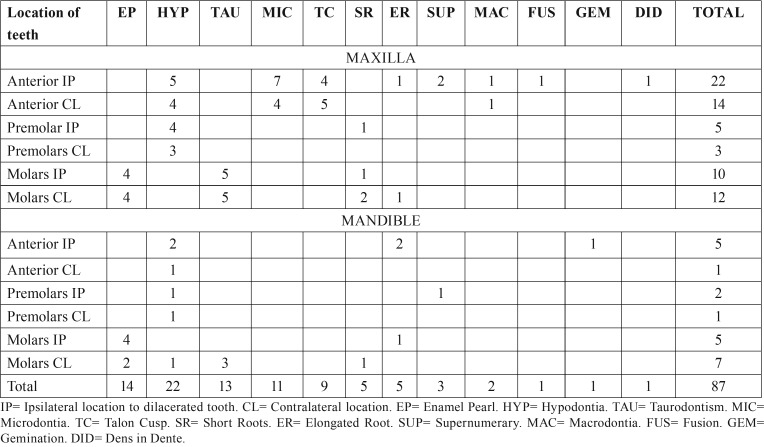
Location of the dental developmental alterations in the studied population.

No clinical or radiographic signs of known syndromes were detected, and analysis of the patient’s clinical histories showed that no parents or relatives were affected with a syndrome or any other genetic disease.

## Discussion

Dilaceration is a well-known DDA involving change in the normal alignment of the root and the crown. Features of this entity are not well understood and present data on its origin, frequency, gender preference and most commonly involved teeth is controversial (4).

Diagnosis of unidentified veiled entities are somewhat problematic, and the panoramic radiograph is a very useful tool to detect clinically hidden lesions, tumors and alterations. Identification of teeth with dilacerated roots is not uncommon during the dental practice, Endodontics and Oral and Maxillofacial Pathology, but the presence of DDAs in patients with dilaceration have received no attention in previous literature and its frequency and pathogenic mechanisms are unknown.

According to Luder (5), root developmental alterations (RDAs) and malformations appear as elongated roots, root dilaceration, cervical mineralized diaphragm (molar-incisor malformation), concrescence, taurodontism and short root anomaly and all of them are associated to arrested root development. Also, other RDAs could develop associated to some inherited diseases: Regional odontodysplasia, hypophosphatasia, dentin dysplasia and others (5). Interestingly, results from animal studies reported in the literature communicated that root alterations were related to defective genes as Nfic (Nuclear Factor I C), Ptc (patched), DKK1 (Dikkopf-related protein 1), Osx (osterix), Smad 4 (mothers against decapentaplegic homolog 4) and Wls (wntless) genes (6-11). Nevertheless, only CLNC7 (osteopetrosis, infantile malignant 2) and PLG (Plasminogen) genes have been reported associated to hereditary root malformations in humans (12,13). According to the above mentioned, it was considered that RDAs are associated to disturbance and/or failure of the epithelial diaphragm during the early root development or to a deficient apical growth in length of the Hertwig’s epithelial root sheath (12,13). Previous studies communicated that root dilaceration can be found in patients with some genetic syndromes as Kabuki, Axenfeld-Rieger, Smith–Magenis Ehlers-Danlos and Proteus (14-18). These patients showed dilacerated teeth and other dental anomalies: tooth agenesis, taurodontism, enamel hypomaturation, microdontia, screwdriver central incisors, peg-shaped lateral incisors, enamel hypoplasia, short roots, atypical furcation of the roots, and supernumerary roots. Also, there are published three isolated cases on simultaneous appearance of dilacerated teeth with supernumeraries, tooth transposition and tooth fusion (19-21).

This is the first report of a large series of cases on the simultaneous occurrence of dilacerated root teeth and different types of DDAs in the same patient. Our review of the literature showed that no clinicopathological studies dealing on this unusual association in any patient population was published. Former studies on DDAs informed that they can be found at different rates, in diverse populations from different countries, but any of them was on the association of two or more DDAs as it is analyzed in this study. Also, any statement in relation with this issue was noticed in these reports. Unexpectedly, in this study we observed that different kinds of DDAs were detected in a very high rate of patients with dilacerated teeth (45%). Based in the results of this study, we can suggest that a careful review of the radiographic material will allow the detection of veiled developmental anomalies. Precise diagnosis and timely intervention in patients with dilaceration and other DDAs could reduce, prevent or eliminate serious complications.

It is important to point out that occurrence of simultaneous DDAs suggests synchronic developmental defects during dental growth and it is suspicious for the existence of a systemic or genomic abnormality.

## Conclusions

45.5% is a very high proportion of DDAs in patients with dilacerated roots. This finding strongly suggests that patients with dilacerated teeth should be carefully screened since many of them could present other DDAs. Simultaneous occurrence of dilaceration and DDAs suggests synchronic developmental defects during dental growth.
